# Supplementary material for: Dynamic Changes in Hepatitis A Immunity in Regions with Different Vaccination Strategies and Different Vaccination Coverage
Source: Vaccines (Basel). 2022 Aug 29;10(9):1423. doi: 10.3390/vaccines10091423 (PMC9506316; doi:10.3390/vaccines10091423)
Supplement: Supplementary file 1 [file vaccines-10-01423-s001.zip › Supplementary table S6.pdf]

Supplementary Table S6. Hepatitis A vaccination coverage in 2014-2021 in studied regions compared to the average in Russia

Region	Year	HAV vaccination coverage					
		**Total number of vaccinated**	**Total population, thousand people**	**Vaccination coverage in total population, per 100,000**	**Total number of vaccinated children under 18 years**	**Child population, thousand people**	**Vaccination coverage in children, per 100,000**
Moscow region	2014	63,926	18,920.5	337.9	23,843	2,927.5	814.3
	2015	67,833	19,134.9	354.5	23,262	3,013.4	771.9
	2016	61,436	19,332.8	317.8	18,744	3,111.0	602.5
	2017	82,904	19,263.8	430.4	16,514	3,229.2	511.4
	2018	112,226	19,726.9	568.9	22,772	3,353.4	679.1
	2019	109,123	20,119.4	542.4	25,892	3,566.4	726.0
	2020	110,520	20,293.0	544.6	37,955	3,651.6	1,039.4
	2021	141,862	20,293.6	699.0	50,730	3,651.6	1,389.3
Sverdlovsk region	2014	117,680	4,311.9	2,792.1	92,344	801.7	11,518.7
	2015	107,876	4,316.8	2,499.0	80,624	816.3	9,876.4
	2016	71,744	4,322.8	1,659.7	49,017	833.3	5,882.0
	2017	55,847	4,328.7	1,290.2	37,608	868.9	4,328.5
	2018	44,914	4,329.6	1,037.4	24,848	891.9	2,786.0
	2019	42,049	4,320.6	973.2	24,482	952.2	2,570.1
	2020	34,545	4,314.0	800.8	19,042	934.1	2,038.5
	2021	35,715	4,314.0	827.9	22,648	934.2	2,424.3
Tuva Republic	2014	15,206	309.9	4,907.4	14,982	108.7	13,783
	2015	8,433	311.1	2,710.6	8,433	110.8	7,613.8
	2016	6,016	312.5	1,925.1	6,016	112.8	5,334.0
	2017	10,244	314.7	3,255.6	10,244	115.0	8,909.6
	2018	8,586	317.1	2,707.7	8,586	117.0	7,337.3
	2019	7,674	321.2	2,389.3	7,661	120.6	6,352.6
	2020	6,932	327.0	2,120.0	6,932	122.0	5,684.2
	2021	6,520	326.1	1,999.5	6,499	121.8	5,336.4
	2014	32,035	955.8	3,351.7	21,521	254.2	8,464.9

Sakha Republic (Yakutia)	2015	20,989	955.1	2,197.6	12,210	254.8	4,792.4
	2016	22,950	955.8	2,401.1	13,235	256.4	5,161.7
	2017	20,449	958.3	2,133.9	11,609	259.6	4,471.7
	2018	18,365	961.2	1,910.5	10,331	262.0	3,943.0
	2019	14,756	965.7	1,528.0	8,437	264.2	3,193.5
	2020	13,030	969.5	1,344.0	8,418	267.8	3,143.5
	2021	12,441	969.5	1,283.2	8,470	264.1	3,206.7
Khabarovsk region	2014	7,916	1,342.8	589.5	7,521	243.9	3,083.6
	2015	1,530	1,341.5	114.1	1,291	246.9	522.8
	2016	3,205	1,338.6	239.4	2,931	252.5	1,161.0
	2017	3,476	1,336,5	260.1	2,973	259.1	1,147.6
	2018	2,458	1,334.0	184.3	2,099	263.1	797.8
	2019	2,460	1,324.9	185.7	1,390	274.6	506.2
	2020	14,490	1,318.3	1,099.1	3,624	277,2	1,307.2
	2021	1,687	1,319.7	127.8	1,024	277.2	369.4
Russian Federation, average	2014	519,857	143,207.3	363.0	321,459	26,722.0	1,203.1
	2015	475,390	145,886.2	325.7	298,003	27,411.8	1,087.1
	2016	427,201	146,063.2	292.5	255,710	27,826.1	919.0
	2017	502,575	146,415.8	343.3	260,222	28,647.1	908.4
	2018	469,605	146,671.5	320.2	162,209	29,294.1	553.7
	2019	504,116	146,793.3	343.4	178,081	30,099.0	591.6
	2020	349,695	146,764.1	238.3	129,435	30,291.7	427.3
	2021	436,993	146,815.7	297.6	181,016	30,295.7	597.5
