# Supplementary material for: Dynamic Changes in Hepatitis A Immunity in Regions with Different Vaccination Strategies and Different Vaccination Coverage
Source: Vaccines (Basel). 2022 Aug 29;10(9):1423. doi: 10.3390/vaccines10091423 (PMC9506316; doi:10.3390/vaccines10091423)
Supplement: Supplementary file 1 [file vaccines-10-01423-s001.zip › Supplementary tables S1-S5.pdf]

Table S1. Age-specific anti-HAV prevalence, Moscow Region

Age group, years	Year	N	N pos (>20 IU/l)	%	95% CI	*P* value*
**<1**	2008	97	24	24.7	17.2 – 34.2	1
	2020	0	-	-	-	
**1-9**	2008	237	31	13.1	9.3 – 18.0	0.7224
	2020	530	64	12.1	9.6 – 15.1	
**10-14**	2008	121	21	17.4	11.6 – 25.2	**0.0042**
	2020	184	12	6.5	3.8 – 11.0	
**15-19**	2008	152	29	19.1	13.6 – 26.1	**0.0009**
	2020	212	15	7.1	4.3 – 11.3	
**20-29**	2008	104	38	36.5	27.9 – 46.1	**>0.0001**
	2020	2012	240	11.9	10.6 – 13.4	
**30-39**	2008	100	41	41.0	31.9 – 50.1	**>0.0001**
	2020	2751	578	21.0	19.5 – 22.6	
**40-49**	2008	105	59	56.2	46.7 – 65.3	**>0.0001**
	2020	1044	379	36.3	33.4 – 39.3	
**50-59**	2008	114	86	75.4	66.8 – 82.5	**>0.0001**
	2020	490	239	48.8	44.4 – 53.2	
**≥60**	2008	149	144	96.6	92.2 – 98.8	**>0.0001**
	2020	269	195	72.5	66.9 – 77.5	

* Fisher's exact test

Table S2. Age-specific anti-HAV prevalence, Sverdlovsk Region

Age group, years	Year	N	N pos (>20 IU/l)	%	95% CI	*P* value*
<1	2008	101	37	36.6	27.9 – 46.4	0.4180
	2020	35	10	28.6	16.2 – 45.2	
1-9	2008	200	138	69.0	62.3 – 75.0	**>0.0001**
	2020	137	29	21.2	15.1 – 28.8	
10-14	2008	120	15	12.5	7.6 – 19.7	**>0.0001**
	2020	61	41	67.2	54.7 – 77.7	
15-19	2008	100	23	23.0	15.8 – 32.2	**>0.0001**
	2020	204	162	79.4	73.3 – 84.4	
20-29	2008	81	32	39.5	29.6 – 50.4	0.1523
	2020	51	27	65.4	54.3 – 75.0	
30-39	2008	106	53	50.0	40.7 – 59.4	0.1232
	2020	147	58	39.5	31.9 – 47.5	
40-49	2008	104	66	63.5	53.9 – 72.1	**0.0247**
	2020	102	48	47.1	37.7 – 56.7	
50-59	2008	107	87	81.3	72.8 – 87.6	**>0.0001**
	2020	99	54	54.6	44.8 – 64.0	
≥60	2008	108	104	96.3	90.6 – 98.9	**0.0011**
	2020	99	81	81.8	73.0 – 88.3	

* Fisher's exact test

Table S3. Age-specific anti-HAV prevalence, Tuva Republic

Age group, years	Year	N	N pos (>20 IU/l)	%	95% CI	*P* value*
<1	2008	88	49	55.7	45.3 - 65.6	**0.0041**
	2020	68	53	77.9	66.6 – 86.3	
1-9	2008	200	70	35.0	28.7 - 41.8	**0.0016**
	2020	169	87	51.5	44.0 – 58.9	
10-14	2008	100	66	66.0	56.3 - 74.6	0.6399
	2020	85	59	69.4	58.9 – 78.2	
15-19	2008	100	86	86.0	77.7 - 91.6	**>0.0001**
	2020	115	71	61.7	52.6 – 70.1	
20-29	2008	100	98	98.0	92.6 – 99.9	**>0.0001**
	2020	70	42	60.0	48.3 – 70.7	
30-39	2008	100	96	96.0	89.8 – 98.8	**0.0355**
	2020	139	122	80.1	73.2 – 86.3	
40-49	2008	100	98	98.0	92.6 – 99.9	0.6749
	2020	94	91	96.8	90.6 – 99.3	
50-59	2008	100	97	97.0	91.2 – 99.4	1
	2020	89	87	97.8	91.7 – 99.9	
≥60	2008	123	122	99.2	95.1 - 100	1
	2020	64	64	100	93.2 - 100	

* Fisher's exact test

Table S4. Age-specific anti-HAV prevalence, Sakha Republic (Yakutia)

**Age group, years**	**Year**	**N**	**N pos (>20 IU/l)**	**%**	**95% CI**	***P* value***
<1	2008	103	23	22.3	15.3 – 31.3	**0.0004**
	2020	58	29	50.0	37.5 – 62.5	
1-9	2008	203	46	22.7	17.4 – 28.9	**>0.0001**
	2020	185	100	54.1	46.7 – 61.1	
10-14	2008	100	30	30.0	21.9 – 39.6	**0.0157**
	2020	109	51	46.8	37.7 – 56.1	
15-19	2008	100	22	22.0	14.9 – 31.1	**>0.0001**
	2020	90	61	67.8	57.5 – 76.6	
20-29	2008	123	61	49.6	40.9 – 58.3	**0.0433**
	2020	101	64	63.4	53.6 – 72.1	
30-39	2008	100	66	66.0	56.3 – 74.6	0.8817
	2020	101	68	67.3	57.7 – 75.7	
40-49	2008	100	79	79.0	70.0 – 85.9	1
	2020	85	68	80.0	70.2 – 87.2	
50-59	2008	99	88	88.9	81.0 – 93.8	0.3328
	2020	101	94	93.1	86.2 – 96.8	
≥60	2008	97	94	96.9	90.9 – 99.3	0.4714
	2020	81	76	93.8	86.0 – 97.7	

* Fisher's exact test

Table S5. Age-specific anti-HAV prevalence, Khabarovsk Region

**Age group, years**	**Year**	**N**	**N pos (>20 IU/l)**	**%**	**95% CI**	***P* value***
<1	2008	100	33	33.0	24.5 – 42.7	1
	2020	0	-	-	-	
1-9	2008	197	25	12.7	8.7 – 18.1	0.3902
	2020	1255	191	15.2	13.3 – 17.3	
10-14	2008	100	28	28.0	20.1 – 37.5	0.9042
	2020	515	150	29.1	25.4 – 33.2	
15-19	2008	99	19	19.2	12.6 – 28.1	0.1319
	2020	527	141	26.8	23.2 – 30.7	
20-29	2008	101	45	44.6	35.2 – 54.3	**>0.0001**
	2020	713	141	19.8	17.0 – 22.9	
30-39	2008	99	57	57.6	47.7 – 66.9	**>0.0001**
	2020	624	201	32.2	28.7 –36.0	
40-49	2008	100	68	68.0	58.3 – 76.4	0.2808
	2020	294	181	61.6	55.9 – 66.9	
50-59	2008	100	62	62.0	52.2 – 70.9	0.0627
	2020	333	240	72.1	67.0 –76.6	
≥60	2008	99	79	79.8	70.8 – 86.6	**0.0322**
	2020	509	450	88.4	85.3 – 90.9	

* Fisher’s exact test
